# Identifying Witnessed Suicides in National Violent Death Reporting System Narratives

**DOI:** 10.3390/healthcare12020209

**Published:** 2024-01-15

**Authors:** Vickie M. Mays, Mikaela Gareeb, Xingruo Zhang, Vivian Nguyen, Joelle Rosenberg, Yuri Lin, Alina Arseniev-Koehler, Adam Eliav, Jacob Gates Foster, Mika Baumgardner, Susan D. Cochran

**Affiliations:** 1 Department of Health Policy and Management, UCLA Fielding School of Public Health, Los Angeles, CA 90095, USA; 2 Department of Psychology, University of California, Los Angeles (UCLA), Los Angeles, CA 90095, USA; mikatheamazing@gmail.com; 3 UCLA BRITE Center for Science, Research and Policy, University of California, Los Angeles (UCLA), Los Angeles, CA 90095, USA; mikaelagareeb@yahoo.com (M.G.); vkcnguyen@g.ucla.edu (V.N.); ylin010101@gmail.com (Y.L.); aaashelm@purdue.edu (A.A.-K.); adam.eliav96@gmail.com (A.E.); foster@soc.ucla.edu (J.G.F.); cochran@ucla.edu (S.D.C.); 4 Department of Public Health Sciences, University of Chicago, Chicago, IL 60637, USA; xrzhang@uchicago.edu; 5 Department of Sociology, University of California, Los Angeles (UCLA), Los Angeles, CA 90095, USA; joellerosenberg@ucla.edu; 6 Department of Sociology, Purdue University, West Lafayette, IN 47907, USA; 7 Department of Biomedical Informatics, University of California, San Diego (UCSD), San Diego, CA 92093, USA; 8 Department of Epidemiology, UCLA Fielding School of Public Health, Los Angeles, CA 90095, USA; 9 Department of Statistics, University of California, Los Angeles (UCLA), Los Angeles, CA 90095, USA

**Keywords:** suicide prevention, coding of suicides, NVDRS, lesbian, gay, bisexual, narratives

## Abstract

There is increasing attention to suicides that occur in view of others, as these deaths can cause significant psychological impact on witnesses. This study illuminates characteristics of witnessed suicides and compares characteristics of these deaths to non-witnessed suicides. We develop a codable definition of what constitutes witnessed (vs. non-witnessed) suicide. Our data include a sample of 1200 suicide descriptions from the 2003–2017 National Violent Death Reporting System (NVDRS). We first developed criteria to identify probable cases of witnessed suicide. The coding scheme achieved 94.5% agreement and identified approximately 10% (*n* = 125) of suicides as witnessed. Next, we examined differences between witnessed and non-witnessed suicides in demographics, manner of death, and social/environmental factors using bivariate Chi-squared tests, multivariate logistic regression, and ANOVA. Witnessed suicide decedents were significantly more likely than non-witnessed suicide decedents to be male, younger, and members of a sexual minority, and to have died in living spaces by means of a firearm. Two thirds of witnesses were strangers to the decedents, while 23.2% were romantic partners or ex-partners of the decedents. Our coding method offers a reliable approach to identify witnessed suicides. While witnessed suicides are relatively infrequent, these deaths have profound impact on witnesses. Articulating the features of witnessed suicides may contribute to identifying potential risk mitigation strategies.

## 1. Introduction

In 2021, there were an estimated 47,646 deaths due to suicide in the United States—a 4% increase from 2020 [1]. It has been estimated that for each suicide, 135 other people are affected by the death, and of those 22% experience subsequent guilt, anger, shock, and depression [2]. Although the majority of suicides occur in the decedent’s home while alone and in private areas (e.g., bedrooms, closets, and bathrooms), increasingly a subset of suicides take place within view of others, such as using a firearm in the presence of other people, jumping in front of trains or subways, or leaping from bridges [3,4]. This has been referred to as witnessed suicide [5,6]. Studies have found that 4–10% of suicides are witnessed [5,6,7,8,9]. Although relatively rare, their impact on witnesses can be profound [10,11,12,13]. Studies documenting the mental health state of “survivor-victims,” or those who have witnessed a suicide, have observed that these individuals commonly experience strong feelings of shame, guilt, and anger [5,6,14]. One study found that survivor-victims “are obsessed with thoughts of death, seeking reasons, casting blame, and often punishing themselves” [15].

Research on witnessed suicides has investigated their likely locations and circumstances, as well as characteristics of decedents and witnesses. For example, one study examined suicides from the Golden Gate Bridge, a widely known and very public location that averages 30 suicides a year [16]. These deaths are likely to be witnessed. Decedents who jumped typically have histories of psychiatric diagnoses including schizophrenia or psychosis [16,17,18]. Railway or subway locations also present another suicide location that is likely to be witnessed. A majority of decedents in railway- or subway-linked suicides have been found to also have previous psychiatric hospitalization and evidenced aberrant behaviors pre-death while on the station platform [19,20,21,22,23].

While witnessed suicides are often traumatic for the observer, they may also offer opportunities for interventions from observers. To that end, articulating common patterns of relationship between decedents and witnesses and identifying likely methods and locations of witnessed successful suicides as well as possible motivations of the decedent could contribute to preventative measures by identifying high risk situations [4]. In the current study, we develop a standardized definition of what constitutes witnessed (vs. non-witnessed suicide). Then, we apply our approach to identify decedent characteristics, location, and relationship to witnesses, employing narratives from the National Violent Death Reporting System (NVDRS). As our work is part of a larger study on identifying sexual orientation and characteristics of suicide in the NVDRS [24,25], we selected our sample, in part, on the NVDRS coded sexual orientation status of the decedent to ensure that our definition would be inclusive of the suicide circumstances of this population.

## 2. Materials and Methods

### 2.1. Data Source

We used information drawn from the 2003–2017 NVDRS. Established by the Centers for Disease Control and Prevention in 2002, the 2003–2017 NVDRS database includes reports from approximately 325,000 violent deaths. Over time, the NVDRS has incorporated reports from an increasing number of states, with 34 reporting in the 2017 year of the database [26]. The NVDRS is a compilation of both close-ended information (e.g., demographic information, decedent characteristics) and potentially two narrative reports written by a trained state-level public health worker, one summarizing law enforcement records and the other coroner/medical examiner files (LE/CME). Narratives can include information such as decedent demographics, place and cause of death, toxicology results, and method of death (see references for more details) [25,27]. This study was exempt from human subjects review.

We first restricted the sample to deaths where the manner of death was determined to be suicide or homicide/suicide (*n* = 203,715). From this, we drew three samples: a systematic sample of 620 LGBT deaths described in Lyons et al. [24]; all deaths coded by the NVDRS as having a lesbian, gay, bisexual, or transgender (LGBT) identity not included in the Lyons et al. sample (*n* = 1028); and then a 10% simple random sample of the remaining suicide deaths (*n* = 20,376). From these deaths, we randomly selected 1200 for study. Coders were blind as to the original sexual orientation coding of the decedent.

### 2.2. Methods for Case Classification of Witnessed Suicide

#### 2.2.1. Definition of Witnessed Suicide

The NVDRS does not code whether a suicide case is witnessed or non-witnessed. To develop criteria for case classification, five coders, all with previous training in suicide prevention, were tasked with developing a coding system. After reading subsets of the study narratives, they derived inclusion and exclusion criteria for case classification. In addition, they developed a coding sheet with examples to guide naïve coders. For this study, they defined a witnessed suicide as any suicide in which the decedent died in a location where an individual or individuals either observed the death as it happened or were highly likely to observe the suicide. Locations open to public access (i.e., commercial buildings, educational institutions, houses of worship, government buildings, healthcare facilities, and unobstructed open areas) were considered public areas with a high probability of individuals witnessing the suicide. Additionally, all cases where the method of death was a jump or fall from public areas, railroads, and subways was deemed a witnessed suicide. Furthermore, deaths arising in the context of Russian roulette games with more than one person were coded as witnessed suicides. Area locations with low likelihood of a witness observing the suicide, such as in the decedent’s own vehicle in a parking lot, obstructed public areas such as alleys, or private office spaces, were not included and would fall into non-witnessed suicides. Unless a witness was explicitly mentioned, all private residential areas and private rooms within public locations (i.e., areas only open to employees or staff, personal offices, closets, etc.) were not coded as witnessed suicide and were classified as non-witnessed cases. Cases in which an individual overheard the suicide occurring, such as a gunshot from a different room or over a phone call, were not included as witnessed but categorized as non-witnessed cases. Lastly, cases in which a witness observed an act of suicide such as swallowing pills were considered to be witnessed suicides.

#### 2.2.2. Coding Procedures and Inter-rater Agreement

To arrive at a reliable coding scheme (see Table 1), narrative cases were randomly distributed in seven separate batches. The first 50 cases in Batch One served as training cases to develop coding criteria. Batch Two contained an additional 50 cases, Batch Three 200 cases, Batch Four 300 cases, Batch Five 98 cases, Batch Six 199 cases, and Batch Seven 203 cases, for a total of *n* = 1200 cases. Coders began with small batches, and then batches were increased in size. Batches sometimes varied based on the selection rubric that ensured cases from the three categories explained above. Each coder used binary coding (0, 1) to classify the presence or absence of witnessed suicide. A four out of five consensus was considered reliable to code the presence/absence of witnessed suicide. Following the coding of each batch, the coders received a report of their agreement/disagreement and reviewed cases with disagreements. Cases where four out of five coders subsequently agreed were deemed consensuses. To strengthen the reliability of the coding, discussions surrounding what classified a highly probably location evolved after discussing Batch Two. For example, locations of low obstruction (e.g., fewer cars, buildings, or trees) may still have a high or low probability of being in view of a witness depending on the foot traffic in that area.

**Figure 1:**
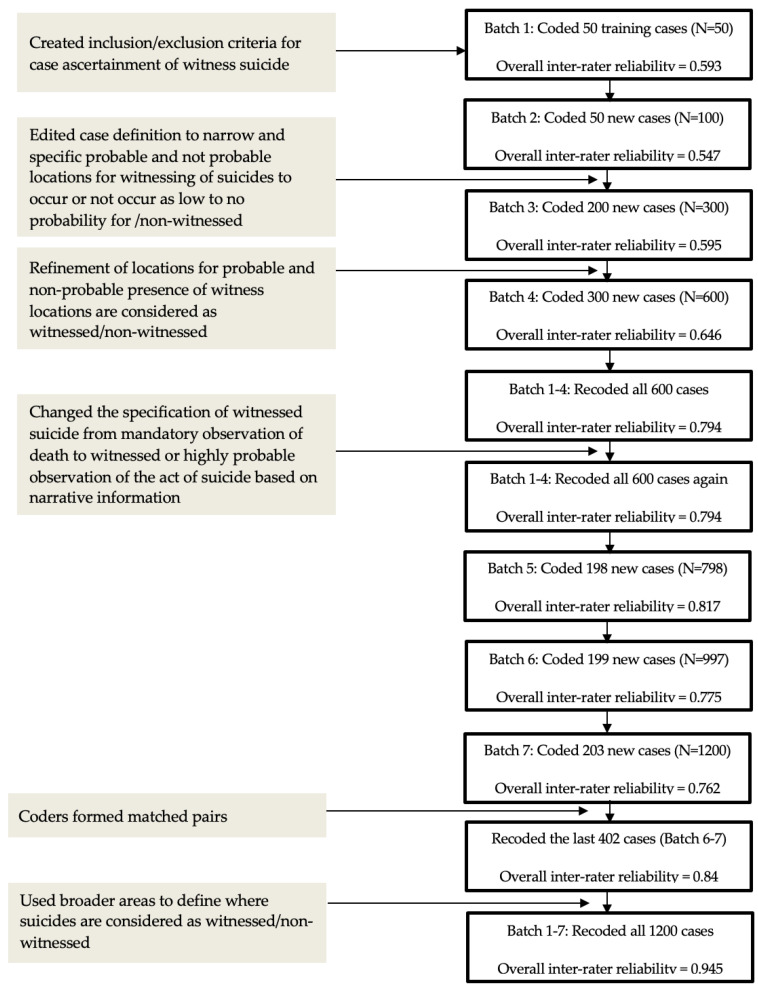
Inter-rater agreement of coding for witnessed suicide using the National Violent Death Reporting System 2003–2017 narratives.

After re-coding Batches One through Four based on refinement of locations, the definition of witnessed suicide was changed from a mandatory observation of the suicide to also include cases in which there was a high probability of observation. After coding all seven batches, the overall inter-rater agreement was still deemed inadequate, so the witnessed suicide locations were broadened to include types of locations from the Federal Bureau of Investigation (FBI) that are used to classify locations of active shooter incidents in the United States [28]. Coder agreement increased with this additional information. After all seven batches were coded, inter-rater agreement for two of the five coders stood out as consistently poor. These two were paired with coders who had high consistencies of coding. In these matched pairs, the inconsistent coder reviewed 25 cases, explained their rationale, and then to listened to the consistent coder’s view of the same case. This process provided the inconsistent coder with feedback and cognitively shifted understanding of the witnessed suicide criteria. Following this entire process, all coders returned to Batches One through Seven to recode any further disagreements; inter-rater reliability for witnessed suicide at this point reached 94.5% agreement. The agreement between the coders was measured using Fleiss’ kappa [29], which is commonly used for categorical outcomes with more than 2 raters. The calculation was performed using the “irr” package in R [30]. Fleiss’ kappa takes values between −1 and 1. Specifically, values between 0.41 and 0.6 are considered as moderate, values between 0.61 and 0.8 are considered as good, and values between 0.81 and 1 are considered as very good. The pattern of coding review is described in Figure 1.

Of 1200 cases coded, 125 deaths were categorized as witnessed suicides. From the NVDRS narratives, we also coded decedents’ sex (male and female), sexual orientation (lesbian, gay, bisexual, heterosexual, and unknown or missing), transgender identity, and age as well as contextual variables including method of death (pills, fall, firearm, impact from vehicle (non-driver), impact from vehicle (driver), carbon monoxide poisoning, hanging, and stabbing/cutting), location of death (places of commerce, government buildings, open space, educational institutions/office spaces, houses of worship, and communal residences), and relationship of witness to the decedent (romantic (ex-)partner, blood relative, friend/acquaintance, and stranger). Narratives in which a witness was not explicitly mentioned, but where there was a probable presence of a witness, were coded as “stranger”.

We then compared our coding of these variables from the narratives to ones included in the NVDRS quantitative data that are coded by public health workers that prepare the files for the CDC. All of the variables above, with the exception of “relationship of the witness to the decedent,” are included in the NVDRS quantitative data file. Agreement between our coding of narratives on the variables above and the public health workers’ quantitative coding ranged from 98–100% with the exception of one variable essential to the witnessed suicide definition. The variable with lowest agreement (88%) was classification of a suicide occurring in an open space. For the analyses below, we use the original NVDRS variables as well as NVDRS public health coders’ designations for decedent’s race/ethnicity, educational attainment, marital status, military service history, and homeless status; whether financial problems appeared to have contributed to the death; whether the death occurred within one month of the decedent being released from or admitted to an institutional setting; suspected alcohol use in the hours preceding the incident; and, for decedents with a toxicology screen, blood alcohol content (BAC) and the use of marijuana and/or opiate drugs (toxicology results, however, are missing in over half of the cases).

#### 2.2.3. Data Analytic Plan

Data analyses were conducted using R 4.0.2 (R Core Team, 2022) to investigate differences between suicides classified as witnessed and non-witnessed [31]. Initially, bivariate frequencies and percentages were computed. Chi-squared tests were used to compare features between witnessed and non-witnessed suicides while Fisher’s exact tests were used for some variables to accommodate sub-sample size(s) smaller than 5. Effect sizes from the Chi-squared tests are reported as Cramer’s V along with the Chi-squared statistics and their *p*-values. Then, a multivariate logistic regression was utilized to predict witness status, using the same variables as in the bivariate analyses as predictors. We report the adjusted odds ratio (OR) and 95% confidence intervals (CI), and we used the Wald Chi-squared test to determine the overall significance of each explanatory variable.

## 3. Results

### 3.1. Decedent Characteristics of Witnessed Suicide

The majority of the suicide decedents were male, between the age of 26 and 64 years, and non-Hispanic white, and about half had finished high school or completed some college (see Table 2). Decedents of witnessed suicide were also more likely to be homeless compared to decedents of non-witnessed suicide (4.0% vs. 0.7%). A common method of death regardless of being a witnessed or non-witnessed suicide was via firearms (42.4% vs. 42.0%). Falling was a prominent method in witnessed suicide (23.2%) vs. 0.2% in non-witnessed suicide. Witnessed suicides were also less likely to occur in living spaces than non-witnessed suicides (44.8% vs. 76.9%) and more likely to occur in public open spaces (33.6% vs. 8.1%). Based on the Cramer’s V values, age categories, race and ethnicity groups, and highest level of education are considered as having statistically significant association with the presence of witnessed suicide.

Generalized Variance Inflation Factor (GVIF) is small for all the predictors in our final logistic regression, indicating no violation of the no-collinearity assumption. Results from the Wald Chi-squared tests indicate that witnessed and non-witnessed suicides differed significantly in decedents’ ages and sexual orientations, and there is marginally significant association shown between highest level of education and witnessed suicide, while controlling for other predictors in the model (see Table 3). Based on the logistic regression results, in comparison to decedents whose highest levels of education were high school or less, decedents who received some college education were less likely to be classified as a witnessed suicide.

Decedents of witnessed suicides and non-witnessed suicides also significantly differed in the primary type of weapon and means involved. Suicides involving falling were more likely to be witnessed as opposed to non-witnessed. Locations of the suicides also varied; witnessed suicides were more likely to occur in public spaces—including open space, supervised public space, and transportation. Such interpretation is made based on the effect sizes, as these particular odds ratios are not statistically significant. Finally, marijuana use was significantly more likely to be present in witnessed suicides as compared to non-witnessed suicides.

#### Additional Characteristics from Narratives

In 42 (33.6%) of the witnessed suicide incidents, the decedents actively sought to suicide in front of a witness, while in the other 83 (66.4%) incidents, the presence of the witness(es) was incidental. Investigation regarding the relationship of witnesses to decedents determined that two thirds of witnesses were strangers to the decedents, while 23.2% were romantic partners or ex-partners. The remainder were relatives or friends/acquaintances.

## 4. Discussion

While witnessed suicides are relatively rare in comparison to non-witnessed suicides, their impact on those who witness the death can be powerful and enduring [4,5,6,15,32]. In the current study, we developed criteria and a reliable coding scheme to classify suicides for being witnessed or not using information available in the law enforcement and medical/coroner narratives contained within the 2003–2017 NVDRS. Using these classifications, we showed that the majority of witnessed suicide decedents are much younger than decedents from non-witnessed suicides. They are also more likely to be classified as lesbian, gay, or bisexual as opposed to no classification for sexual orientation status. Our deliberate inclusion of LGBT cases may provide some insights into the necessity for further research on sexual orientation as a risk indicator for witnessed suicide. The Interpersonal Theory of Suicide asserts that there are three criteria that must be present for one to have a heightened risk of suicide: (1) perceived burdensomeness, (2) thwarted belongingness, and (3) capability for suicide [33]. Previous studies have demonstrated that sexual minorities feel more isolated than their heterosexual counterparts, which meets the element for thwarted belongingness [34]. Additionally, in both the McDowell et al. (1994) and Ooi et al. (2020) studies, perceived abandonment/isolation and use of firearms were significant factors in witnessed suicides [4,8]. As such, not only does the isolation reported by LGBT individuals increase their risk of suicide, but it may also increase their risk of witnessed suicide, specifically. It is also the case that young LGBT individuals may be more likely to be at risk for homelessness or transient living arrangements particularly in early years of coming out, which may also contribute to witnessed suicide occurrence [35]. Further research would be necessary to confirm this possibility [36]. Given the discrimination and rejection experienced by LGBT populations [34,37,38], further explorations of this theme warrant attention.

Homelessness, falls, public open spaces, and transportation are all risk factors for witnessed suicide because the element that ties these disparate matters together is the fact that they inherently occur where others are likely to see them. Similarly, falling from heights, being in public open spaces, and driving a vehicle occur in locations where there is a high probability of being seen by another person.

Suicide via firearm was the most likely method for both witnessed and unwitnessed suicides. However, suicide through jumping or falling was the second most likely method of death for witnessed suicides, and the use of vehicle-related means was the third most likely method to be used. This differs from national statistics, which indicate the second and third most likely means of suicide-related deaths are suffocation and drug poisoning, respectively [1,39,40].

While the NVDRS narratives are somewhat limited in their description of witnesses and the relationship of witnesses to the decedent, previous studies have found that romantic partners are the most likely witnesses to a suicide [5,8]. Here, however, we found the majority of witnesses of NVDRS suicides were strangers, followed by romantic partners or ex-partners. This divergence could be due to our definition of witnessed suicide, which includes probable witnesses, as compared to existing studies, which specify only recorded witnesses. Our focus on locations where witnessed suicides are likely to occur—bridges, trains, and subway stations—may have increased the prevalence of strangers witnessing these deaths. Train and subway conductors, metal workers who attempt bridge rescues, and hotel staff for example are accidental bystanders. Location of suicide is an overlooked variable in suicidology, but yet it offers some insights into occupations of rescuers who are likely to be exposed to witnessed suicides [41,42]. Knowledge of locations in suicides increases knowledge of lethality and risk of being an unintended witness [43,44]. Studies have documented the impact of suicide in occupations such as first responders, mental health professionals, and others [45,46], but less attention has been paid to inadvertent bystanders who by virtue of their occupations may be exposed to the emotional distress of witnessing a suicide. There is a growing focus by suicide prevention and response agencies to “witness survivors” [47,48] and by suicide researchers seeking to address the mental health consequences of suicides [49,50], as well as public awareness of those who have had this experience and its impact on their mental health [51,52]. All three of these groups call for the need for greater attention to “suicide witness survivors”.

Results of our study also provide a case definition that can be applied to what a witnessed vs. non-witnessed suicide is, allowing researchers’ consistency in research approaches on the topic. Our coding system, if employed by large databases such as violent deaths at the local, state, or federal levels, could aid in identifying circumstances of particular occupations that may require specific training and resources to mitigate mental health consequences. In addition, following the efforts of New Hampshire’s Connect program identifying ways to reach those individuals who may be witnesses to get them into treatment if needed could be beneficial [47].

Results from this study should be contextualized by some of its limitations. First, only 1200 cases were selected for study on the assumption that witnessed suicide characteristics are randomly distributed and common in the data. A larger sample may have identified additional characteristics. Additionally, witnessed suicide is a relatively under-studied topic; thus, definitions of witnessed suicide are not well established. Compared to existing definitions, our definition of witnessed suicide includes locations identified as highly likely for a witness to be present and excludes non-visual forms of witnessing (e.g., overhearing a gunshot, being present via phone, etc.) [4,8]. These differences in the definition could impact the results and, subsequently, the implications and knowledge drawn from them. An additional limitation of the study is the NVDRS narratives themselves; the narratives are often short, terse, or nondescriptive of many death circumstances. Thus, our study coders coded conservatively. Despite these limitations, we were able to produce a coding schema with good inter-rater agreement that can identify cases of witnessed suicide from death narratives. Our inclusion of a subsample of LGBT individuals has verified that this approach can work with this vulnerable population.

## 5. Conclusions

In sum, investigating the nature of witnessed suicide and documenting its frequency and associated characteristics is possible given better methods for classifying these deaths. Identifying locations and occupations in which there are unintended mental health consequences from witnessed suicides provides an opportunity to address the risk for particular occupations [43]. In this study, we tested and developed a definition as well as reliable methods for coding witnessed suicides in the NVDRS. These coding criteria can be integrated into public health mortality surveillance systems. As the United States tries to reach its goal of zero suicides, it is important that we not overlook any methods for improvement of the use of suicide data that can decrease suicide and its associated morbidity.

## Figures and Tables

**Table 1:** Definition and coding for witnessed suicide using the NVDRS data 2003–2017.

[Witnessed/Non-Witnessed Suicide]	
Code type	(1, Witnessed Suicide) (0, Non-Witnessed Suicide)
Definition	A witnessed suicide occurs when the victim (V) suicides in a location where an individual or individuals see(s), or is highly likely to see, the V suicide.
When to code presence of [witnessed suicide]	Suicide was mentioned to be observed by others. Location/manner of death suggests that one or more people had a high probability of observing the V suicide.
Examples of PRESENCE of [witnessed suicide]	- “Thirty-seven-year-old male died as the result of serious head injuries when he purposefully jumped off a freeway overpass and was struck by an oncoming RV.” - “Witnessed to use a gun to self-inflict a gunshot to the chest. V was parked in vehicle on the side of a highway road. When a fellow worker noticed the vehicle parked on the side of the freeway and walked up to it, he witnessed the shooting.” - “A young adult male age 19 died as a result of an intentional gunshot wound to his right temple…his fiancé witnessed the shooting”. - “Hotel security were called because a young girl was on the balcony standing dangerously close to the edge. They witnessed the V step forward and again at which point she jumped.” - “The V roommate saw her grab a bottle of pills and pour them into her mouth and shouted that she was going to kill herself.”
When to code absence of [witnessed suicide—case of non-witnessed]	Mention of finding the V after a prolonged period of time after suicide. Any mention of hearing the act or being in close proximity without actually observing the V commit suicide.
Examples of ABSENCE of [witnessed suicide—case of non-witnessed]	Decedent was already deceased: - “39 yo Hispanic female was found dead.” Decedent’s death was heard but not explicitly seen: - “Family in the house heard a gun go off in the shed outside, found the V, and called local law enforcement.” Decedent was found after a long period of time: - “Volunteers on a search and locate mission spotted a body in the woods with both an entry and exit wound in the skull, along with a military issued gun nearby.” Not high probability location and already deceased: - “V (a 53-year-old White male) was found dead in the alley behind a liquor store by a store employee.”
Specific notes: Non-witnessed: Suicide occurs where another individual cannot, or has a low likelihood, of seeing the event take place, regardless of whether or not others can hear the suicide. Witnessed suicide does not have to be an abrupt death like jumping off a building, walking into traffic, or self-inflicted gunshot wounds. Overdosing, drinking poison, etc., in front of another person qualifies as a witnessed suicide. When coding for witnessed suicide, ask: “Did the V suicide in a location where there is a high possibility of being seen by another individual(s)?” regardless of whether the narrative states the suicide was unwitnessed. Witnessed Suicide Location Criteria Commerce Presence: Areas open to the public Absence: Areas open only to employees Education Presence: Areas open to the public Absence: Private rooms House of Worship Presence: Areas open to the public Absence: Areas only open to employees Open Space Presence: High exposure/unobstructed Absence: Low exposure/obstructed Government Buildings Presence: Areas open to the public Absence: Areas only open to employees Residence Non-communal space Presence: Another person is reported to have seen the suicide Absence: No witnesses reported Communal space Presence: Location exposed to shared areas Absence: Unexposed to shared areas Healthcare Presence: Areas open to the public Absence: Private rooms	

Note: V refers to the suicide decedent. Consistent with the CDC’s NVDRS data-use restrictions, all example narratives are fictional but similar to actual NVDRS case descriptions.

**Table 2:** Characteristics by presence/absence of witnessed suicide for quantitative data from the 2003–2017 National Violent Death Reporting System (NVDRS) sample (*n* = 1200).

	Witnessed Suicide (*n* = 125)		Non-Witnessed Suicide (*n* = 1075)		Cramer’s V	Chi-Squared Statistic	*p*-Value
	Frequency	Percent (%)	Frequency	Percent (%)			
Sex							
Male	92	73.6	782	72.7	0.006	0.009	0.922
Female	33	26.4	293	27.3			
Age ***							
0–17	7	5.6	60	5.6	0.128	19.503	<0.001
18–25	24	19.2	172	16.0			
26–44	62	49.6	356	33.1			
45–64	24	19.2	359	33.4			
65+	8	6.4	128	11.9			
Sexual orientation							
Straight/heterosexual	6	4.8	47	4.4	0.069	5.741	0.057
Gay/lesbian/bisexual	47	37.6	297	27.6			
Unknown	72	57.6	731	68.0			
Transgender							
Yes	5	4.0	48	4.5	0.007	< 0.001	0.992
No	120	96.0	1027	95.5			
Race and ethnicity *							
White, non-Hispanic	92	73.6	888	82.6	0.081	7.927	0.048
Black or African American, non-Hispanic	12	9.6	63	5.9			
Hispanic	14	11.2	66	6.1			
Other	7	5.6	58	5.4			
Highest level of education							
High school or less	56	44.8	420	39.1	0.057	3.881	0.422
Some college	25	20.0	223	20.7			
Four-year college degree	7	5.6	104	9.7			
Graduate study	8	6.4	51	4.7			
Unknown	29	23.2	277	25.8			
Marital status							
Never married	74	59.2	522	48.6	-	-	0.158
Married/civil union/domestic partnership	25	20.0	268	24.9			
Formerly married (Widowed/divorced/married, but separated)	24	19.2	247	23.0			
Unknown/single not otherwise specified	2	1.6	38	3.5			
Ever served in the U.S. armed forces							
Yes	10	8.0	156	14.5	0.058	3.456	0.063
No/unknown	115	92.0	919	85.5			
Homeless **							
Yes	5	4.0	8	0.7	0.096	8.247	0.004
No/unknown	120	96.0	1067	99.3			
Recently released from or admitted to an institutional setting (added to NVDRS in August 2013)							
Yes	8	6.4	50	4.6	0.025	0.413	0.521
No/unknown	117	93.6	1025	95.4			
Primary weapon type/means ***							
Various methods	40	32.0	408	38.0	-	-	<0.001
Fall	29	23.2	2	0.2			
Gun-related	53	42.4	452	42.0			
Poisoning	3	2.4	213	19.8			
Location type ***							
Commercial space of businesses	4	3.2	45	4.2	-	-	<0.001
Living space	56	44.8	827	76.9			
Public, open space	42	33.6	87	8.1			
Supervised public space	5	4.0	23	2.1			
Transportation	14	11.2	40	3.7			
Other/unknown	4	3.2	53	4.9			
Alcohol use suspected							
Yes	31	24.8	236	22.0	0.021	0.373	0.542
No/not applicable/unknown	94	75.2	839	78.0			
Blood alcohol content							
0.08% or above	21	16.8	139	12.9	0.045	2.389	0.303
Between 0.01% & 0.08%	11	8.8	73	6.8			
Below the detection limit of the test (<0.01% or non-detectable/not applicable, no testing/unknown	93	74.4	863	80.28			
Marijuana result *							
Present	14	11.2	63	5.9	0.067	4.465	0.035
Not present/not applicable/unknown	111	88.8	1012	94.1			
Opiate result							
Present	9	7.2	134	12.5	0.050	2.477	0.116
Not present/not applicable/unknown	116	92.8	941	87.5			
Financial problems appear to have contributed to the death							
Yes	8	6.4	120	11.2	0.047	2.189	0.139
No	117	93.6	955	88.8			

Note: Bivariate comparisons evaluated using Chi-squared test. * *p* < 0.05; ** *p* < 0.01; *** *p* < 0.001.

**Table 3:** Predictors of witnessed suicide deaths in the 2003–2017 National Violent Death Reporting System (NVDRS) sample (*n* = 1200). The overall significance of each predictor was determined using ANOVA.

Predictor	AOR	95% CI
Sex		
Male	-	-
Female	0.95	0.55–1.66
Age *		
0–17	-	-
18–25	0.73	0.25–2.11
26–44	1.06	0.38–3.00
45–64	0.35	0.11–1.15
65+	0.54	0.14–2.15
Sexual orientation **		
Unknown/not coded	-	-
Gay/lesbian/bisexual	2.27	1.30–3.97
Straight/heterosexual	2.51	0.89–7.05
Transgender		
No	-	-
Yes	0.61	0.17–2.12
Race and ethnicity		
White, non-Hispanic	-	-
Black or African American, non-Hispanic	1.51	0.67–3.42
Hispanic	1.51	0.68–3.37
Other	0.89	0.31–2.55
Highest level of education		
High school or less	-	-
Some college	0.49	0.24–0.99
Four-year college degree	0.36	0.12–1.08
Graduate study	1.55	0.57–4.22
Unknown	0.87	0.48–1.59
Marital status		
Never married	-	-
Married/civil union/domestic partnership	1.48	0.75–2.92
Formerly married (widowed/divorced/married, but separated)	1.45	0.73–2.90
Unknown/single not otherwise specified	0.46	0.09–2.33
Ever served in the U.S. Armed Forces		
No/unknown	-	-
Yes	0.74	0.33–1.67
Homeless **		
No/unknown	-	-
Yes	8.01	1.94–33.09
Recently released from or admitted to an institutional setting (added August 2013)		
No/unknown	-	-
Yes	1.05	0.37–2.97
Primary weapon type/means ***		
Various methods	-	-
Fall	323.04	62.08–1680.83
Gun-related	1.61	0.95–2.74
Poisoning	0.15	0.04–0.54
Location type ***		
Commercial space of businesses		
Living space	0.93	0.22–3.99
Public, open space	8.41	1.88–37.62
Supervised public space	3.72	0.56–24.87
Transportation	6.60	1.28–33.99
Other/unknown	0.87	0.13–5.97
Alcohol use suspected		
No/not applicable/unknown	-	-
Yes	1.45	0.74–2.82
Blood alcohol content		
Below the detection limit of the test (<0.01% or Non-detectable/not applicable, no testing/unknown	-	-
Between 0.01–0.08%	1.53	0.62–3.73
0.08% or above	1.75	0.80–3.81
Marijuana result **		
Not present/not applicable/unknown	-	-
Present	3.41	1.58–7.36
Opiate result		
Not present/not applicable/unknown	-	-
Present	0.78	0.29–2.06
Financial problems appear to have contributed to the death		
No	-	-
Yes	0.55	0.22–1.34

Note: Differences evaluated using logistic regression methods regressing suicide type (witnessed vs. not witnessed) on predictors. AOR (adjusted odds ratio) = adjusting for all other predictors in the model, for per unit increment in; 95% CI (confidence interval) for the estimated adjusted OR. * *p* ≤ 0.05; ** *p* ≤ 0.01; *** *p* ≤ 0.001.

## Data Availability

Data for this study comes from the National Violent Death Reporting System Restricted Access Database (RAD), which is accessible to eligible researchers through agreement with the Centers for Disease Control and Prevention’s National Center for Injury Prevention and Control.
